# Impact of Nanoparticle Consolidation on Charge Separation Efficiency in Anatase TiO_2_ Films

**DOI:** 10.3389/fchem.2021.772116

**Published:** 2021-11-02

**Authors:** Karin Rettenmaier, Thomas Berger

**Affiliations:** Department of Chemistry and Physics of Materials, University of Salzburg, Salzburg, Austria

**Keywords:** nanoparticle films, grain boundaries, deep traps, charge separation, recombination, conductivity, photoelectrochemistry

## Abstract

Mesoporous films and electrodes were prepared from aqueous slurries of isolated anatase TiO_2_ nanoparticles. The resulting layers were annealed in air at temperatures 100°C ≤ *T* ≤ 450°C upon preservation of internal surface area, crystallite size and particle size. The impact of processing temperature on charge separation efficiency in nanoparticle electrodes was tracked *via* photocurrent measurements in the presence of methanol as a hole acceptor. Thermal annealing leads to an increase of the saturated photocurrent and thus of the charge separation efficiency at positive potentials. Furthermore, a shift of capacitive peaks in the cyclic voltammograms of the nanoparticle electrodes points to the modification of the energy of deep traps. Population of these traps triggers recombination possibly due to the action of local electrostatic fields attracting photogenerated holes. Consequently, photocurrents saturate at potentials, at which deep traps are mostly depopulated. Charge separation efficiency was furthermore investigated for nanoparticle films and was tracked via the decomposition of hydrogen peroxide. Our observations evidence an increase of charge separation efficiency upon thermal annealing. The effect of particle consolidation, which we associate with minute atomic rearrangements at particle/particle contacts, is attributed to the energetic modification of deep traps and corresponding modifications of charge transport and recombination, respectively.

## Introduction

The efficient exploitation of photogenerated charge carriers at a photocatalyst surface requires fast charge carrier transport at low recombination rates (Diebold, 2003; Carp et al., 2004; Thompson and Yates, 2006; Fujishima et al., 2008; Henderson, 2011; Schneider et al., 2014). Transport, recombination as well as interfacial transfer are strongly influenced by charge carrier trapping at band gap states in surface and subsurface regions of the photocatalyst (Berger et al., 2016; Liu et al., 2019). In aggregated particle systems, carrier transport (Tirosh et al., 2006; Bian et al., 2012; Wallace and McKenna, 2014; Quirk et al., 2019; Rettenmaier et al., 2019) and recombination (Siedl et al., 2009; Lana-Villarreal et al., 2010; Zhang et al., 2014; Jiménez et al., 2016; Hesari et al., 2019) are impacted furthermore by trap states at solid-solid interfaces corresponding to particle/particle contacts. On the other hand, aggregation leads to the formation of extended photocatalyst structures promoting charge carrier separation (Wang et al., 2006; Lakshminarasimhan et al., 2008; Bian et al., 2012; Park et al., 2013; Wang et al., 2018), which is especially challenging in nanoparticle-based systems due to the absence of significant internal electric fields. The beneficial effect of nanoparticle aggregation on charge separation efficiency has been attributed to the creation of additional pathways involving interparticle charge transfer, which leads to a spatial decoupling of light absorption and interfacial charge transfer. Importantly, associated cooperative effects have been reported both for well-aligned nanocrystal superstructures and for random nanoparticle aggregates. For colloidal dispersions, related studies are complicated by aggregation-induced changes of the optical sample properties (Egerton, 2014). More specifically, particle aggregation or agglomeration may induce significant changes of the ability of colloidal dispersions to absorb and scatter impinging photons. Furthermore, a light-induced inhibition of aggregation (Luo et al., 2017) or even de-aggregation (Mendive et al., 2011) of particles upon photocatalyst operation and an associated increase of the reactive surface area have been claimed. A disentanglement of different contributions (e.g., the cooperative effect in nanoparticle aggregates, changes of the optical properties, or of the active surface area) to aggregation- or agglomeration-induced changes of the photocatalytic properties of particle dispersions is therefore challenging. Indeed, depending on experimental conditions beneficial as well as detrimental effects of particle aggregation / agglomeration on photocatalytic activity have been reported (Wang et al., 2006; Lakshminarasimhan et al., 2008; Park et al., 2013; Egerton, 2014; Pellegrino et al., 2017).

**GRAPHICAL ABSTRACT:**
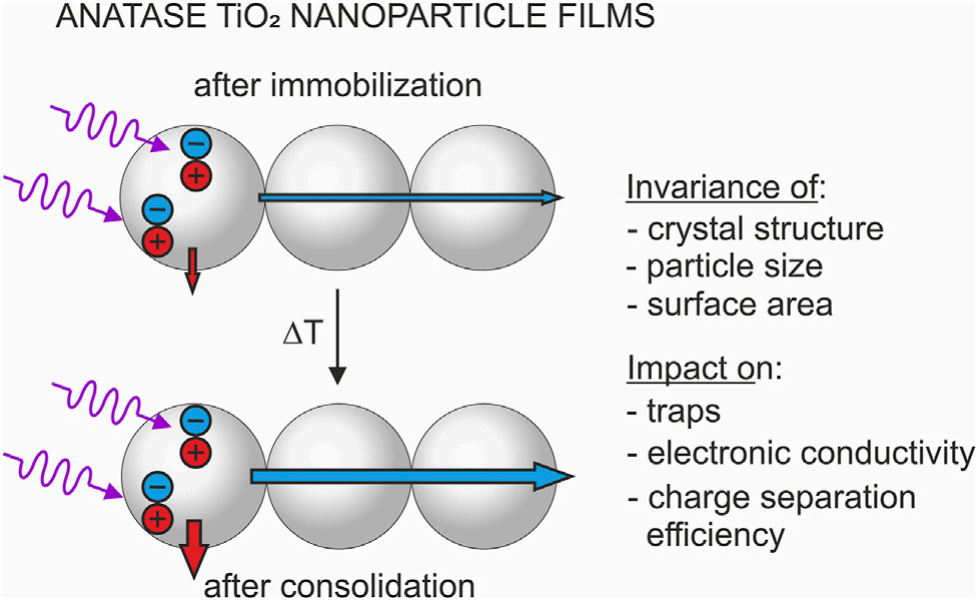


An elucidation of the impact of particle consolidation on the photoactivity therefore requires the use of suitable model systems providing invariable optical properties and constant active surface areas. For this purpose, we immobilized random anatase TiO_2_ nanoparticle networks from aqueous particle slurries onto uncoated glass substrates (for the preparation of nanoparticle films) or onto FTO (fluorine-doped tin oxide)—coated glass substrates (for the preparation of nanoparticle electrodes). The precursor powder used for slurry preparation was synthesized by metal organic chemical vapor synthesis (MOCVS) yielding isolated nanoparticles (Berger et al., 2005). The resulting layers were dried (“pristine films / electrodes”) and annealed in air at temperatures 100°C ≤ *T* ≤ 450°C (“annealed films / electrodes”) pursuing different degrees of particle consolidation. We have shown in a recent study (Rettenmaier et al., 2019) by means of transmission electron microscopy (TEM), X-ray diffraction (XRD) and nitrogen sorption experiments that the original particle size, the crystallite domain size and the specific surface area are preserved for these layers upon thermal processing at *T* ≤ 450°C. However, as revealed by cyclic voltammetry and impedance spectroscopy, thermal annealing leads to a significant modification of the energetic distribution of deep trap states, which were associated with particle/particle contacts (Jankulovska et al., 2012; Rettenmaier et al., 2019). The population of these deep traps was found to strongly affect the electronic conductivity, which experiences an increase by up to five orders of magnitude (Rettenmaier et al., 2019). At positive potentials, pristine as well as annealed films feature a low conductivity. However, at the onset potentials of deep trap population, the conductivity increases exponentially and reaches a saturation value as soon as all deep trap states are filled. At very negative potentials, an annealing temperature-independent conductivity was observed for all electrodes. Thermal annealing leads to a shift of the capacitive peaks associated with deep traps and thus to a shift of the onset potential of exponential conductivity increase. Correspondingly, films annealed at higher temperatures feature a higher electronic conductivity at least under experimental conditions, where deep traps are only partially populated (Rettenmaier et al., 2019).

In the present study, we used these anatase TiO_2_ nanoparticle layers to elucidate the impact of annealing temperature on the separation efficiency of photogenerated charge carriers. As a measure for the charge separation efficiency in nanoparticle electrodes and films, respectively, we measured the photocurrent detected in the presence of methanol (a frequenly employed hole scavenger in photoelectrochemical studies) (Jiang et al., 2001; Monllor-Satoca et al., 2011; Mesa et al., 2017) as well as the quantum yield for the decomposition of H_2_O_2_ (used in photocatalysis as an electron acceptor to increase the degradation rate) (Friedmann et al., 2010).

## Materials and Methods

### Preparation of Nanoparticle Electrodes and Films

The anatase TiO_2_ nanoparticle powder was prepared by metal organic chemical vapor synthesis (MOCVS) (Berger et al., 2005; Elser et al., 2006; Elser and Diwald, 2012). Specifically, titanium (IV)isopropoxide was decomposed at *T* = 800°C in a hot wall reactor system. For purification, the obtained powder samples were subjected to thermal treatment under high vacuum conditions (*p* < 10^−5^ mbar). First, the powder sample was heated to *T* = 600°C using a rate of *r* ≤ 5°C min^−1^. Subsequent oxidation with O_2_ at this temperature was applied to remove organic remnants from the precursor material and to guarantee the stoichiometric composition of the oxide (Berger et al., 2005; Elser et al., 2006; Elser and Diwald, 2012). Resulting powders are characterized by a very low degree of particle aggregation thus yielding an ensemble of virtually isolated nanoparticles (Elser et al., 2006). The powder (0.150 g) was ground in ultrapure water (Milipore, 18.2 MΩ cm, 1.30 ml) in the absence of any additives to avoid the adsorption of organic molecules on the high surface area material (Rettenmaier et al., 2019). The slurry was spread by doctor blading onto FTO-coated glass (Pilkington TEC 8, resistance 8 Ω/□) to obtain anatase TiO_2_ nanoparticle electrodes (“pristine electrodes”). Subsequently, the dried layers were annealed at 100°C ≤ *T* ≤ 450°C for *t* = 1 h in air (“annealed electrodes”) yielding films with a thickness of ∼5 µm (Rettenmaier et al., 2019). As the spreading of the slurry leads to unavoidable variations in the amount of immobilized oxide material, some measurements were conducted using the same electrode for a series of measurements. In these cases, the electrode was thoroughly rinsed with ultrapure water after each measurement, dried in air and annealed repeatedly at temperatures between 100 and 450°C. Importantly, for both processing / characterization approaches the same trends were observed. As confirmed in a previous study (Rettenmaier et al., 2019) using X-ray diffraction, transmission electron microscopy (TEM) and nitrogen sorption, the crystal structure (anatase TiO_2_), the specific surface area (∼90 m^2^/g) and the nanocrystal size are preserved upon annealing of the TiO_2_ films at 100°C ≤ *T* ≤ 450°C. In-depth analysis (Rettenmaier et al., 2019) yields for both films a narrow particle size distribution with a median particle diameter of 14.5 ± 0.5 nm. Accordingly, observed variations in the charge separation efficiency can be attributed to annealing-induced interface formation between particles featuring invariant primary particle properties.

### Cyclic Voltammetry

Cyclic voltammograms of pristine and annealed anatase TiO_2_ nanoparticle electrodes were recorded with a scan rate *v* = 0.020 V s^−1^ in the potential window −0.6 V ≤ *E*_Ag/AgCl_ ≤ + 0.6 V in N_2_-purged 1 M MeOH/0.1 M HClO_4_ aqueous solution. Perchloric acid (HClO_4_, 70% w/w in water) and methanol (MeOH, ≥ 99.8%) were purchased from Sigma Aldrich and used without further purification. The electrochemically accumulated charge was determined from the cathodic (i.e., negative-going) scan. For photoelectrochemical measurements the electrodes were illuminated from the electrolyte side with polychromatic light (*P* = 500 mW cm^−2^) of a 300 W Xe discharge arc lamp (LOT QuantumDesign) equipped with a water filter.

### Hydrogen Peroxide Decomposition

Anatase TiO_2_ nanoparticle films (*A* = 1.82 ± 0.05 cm^2^, *m* = 1.5 ± 0.2 mg) were deposited onto microscopy glass slides and were annealed at *T* = 100°C and *T* = 450°C in air. The mass of the oxide was determined gravimetrically. For all experiments, a freshly prepared film was immersed into a 4.0 mM H_2_O_2_ aqueous solution (*V* = 100.0 ml, Sigma Aldrich, 30 wt%, containing inhibitor). TiO_2_ films were photoexcited by monochromatic light (*λ* = 360 nm) with an irradiance of *P* = 10 mW cm^−2^ corresponding to ≈3.3∙10^16^ photons per second. For this purpose a 1000 W Xe discharge arc lamp (LOT QuantumDesign) equipped with a water filter and a 360 nm band pass filter (FWHM = 40 nm, 38% transmission) was used. Both, photogenerated electrons and holes can react with H_2_O_2_ to form H_2_O and O_2_ (Hirakawa and Nosaka, 2002). To avoid thermal H_2_O_2_ decomposition due to sample heating upon UV exposure, the reaction batch was cooled by an ice bath. In addition, the aqueous H_2_O_2_ solution was continuously stirred to avoid concentration and temperature gradients. Blind tests were performed using blank glass substrates instead of TiO_2_-covered substrates. The H_2_O_2_ concentration of the solution (*V* = 100 ml) before [C(H_2_O_2_) ∼ 4 mM, corresponding to a total of 2.4∙10^20^ H_2_O_2_ molecules] and after the exposure to UV light was determined by KMnO_4_ titration (Huckaba and Keyes, 1948; Nishimura et al., 2008). The titration consisted of

• adding 10 ml H_2_SO_4_ (Merck, for analysis EMSURE, 25%) to 25 ml of analyte (H_2_O_2_ solution),
• dilution of the acidic analyte to *V* = 100 ml with ultrapure water,
• titration of the analyte using 0.02 M KMnO_4_ solution (Merck, Titripur) as titrator

From the photon energy (*E*_ph_ = 3.44 eV, corresponding to *λ* = 360 nm) and the light irradiance (*P* = 10 mW cm^−2^ = 6.242∙10^16^ eVs^−1^ cm^−2^) the incident photon flux (*Φ*_q_) was calculated. The apparent quantum yield (*Φ*) of H_2_O_2_ decomposition on TiO_2_ films annealed at 100 and 450°C was calculated from the incident photon flux (*Φ*_q_) and the number of H_2_O_2_ molecules decomposed upon UV exposure $\left(N_{H_{2}O_{2}}\right)$ according to

$$\Phi =\frac{N_{H_{2}O_{2}}}{\Phi_{q}\cdot t}\cdot 100\%$$

## Results and Discussion

### Electrochemical Charge Accumulation

Prior to charge separation experiments, all electrodes were characterized (in the absence of UV light) by cyclic voltammetry. The results resemble observations from our previous study (Rettenmaier et al., 2019). As they set the base for charge separation experiments, they will be shortly discussed in the following.

**FIGURE 1:**
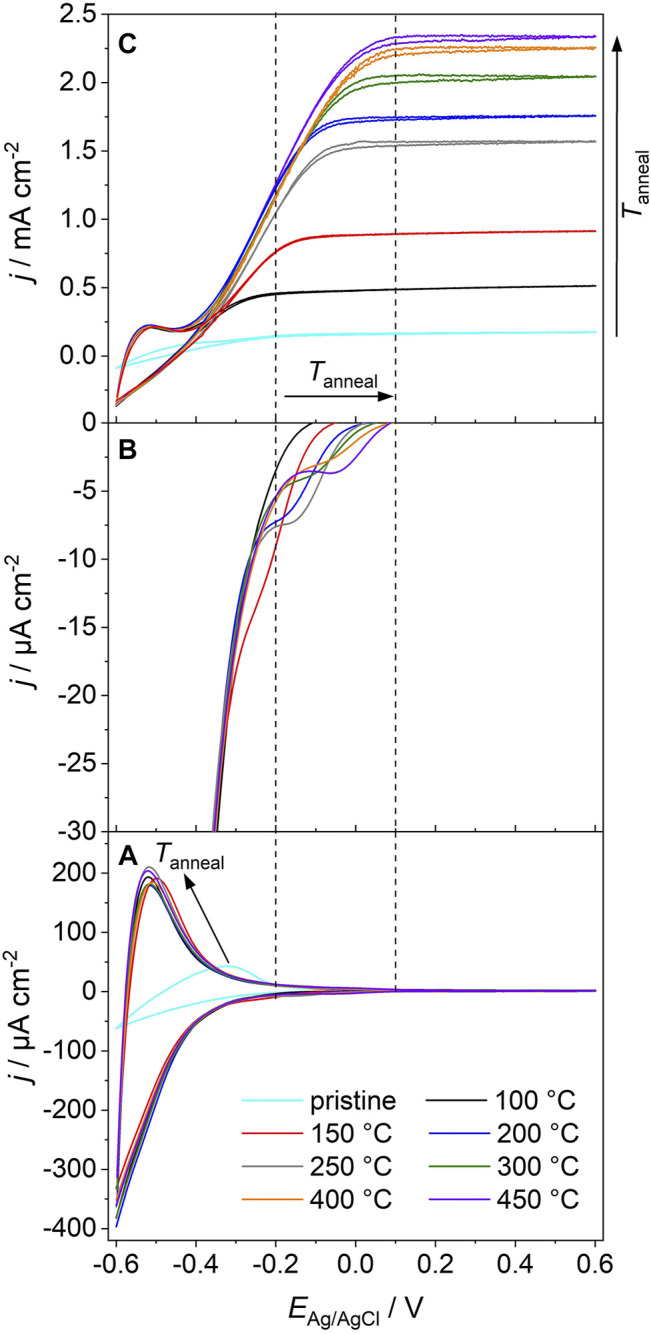
**(A)** Cyclic voltammograms (measured in the absence of UV light) of a pristine anatase TiO_2_ nanoparticle electrode and of an electrode, which was sequentially annealed at 100°C ≤ *T*_*anneal*_ ≤ 450°C. **(B)** Amplification of capacitive currents in the potential range −0.35 V ≤ *E* ≤ 0.1 V [corresponding to the cathodic scans in **(A)**] for the annealed electrodes. **(C)** Cyclic voltammograms of the pristine and the annealed electrodes as recorded upon electrode exposure to polychromatic UV/Vis light (*P* = 500 mW cm^−2^). Electrolyte: N_2_-purged 1 M MeOH/0.1 M HClO_4_ aqueous solution; scan rate: *v* = 0.020 V s^−1^. The vertical lines are a guide to the eye and indicate the electrode potentials at which deep traps are depopulated (in the absence of UV light) on electrodes annealed at 100 and 450°C, respectively.

The cyclic voltammograms (CVs) of a pristine film and of films annealed at different temperatures feature—in the absence of electron acceptors in the electrolyte—capacitive currents at potentials −0.6 V < *E*_Ag/AgCl_ < 0.2 V (Figure 1A) in line with previous studies (Fabregat-Santiago et al., 2003; Bisquert et al., 2008; Berger et al., 2012a; Bertoluzzi et al., 2013). These currents are associated with the population and depopulation of band gap states at solid/electrolyte and solid/solid interfaces in the semiconductor film (Bisquert et al., 2008; Berger et al., 2012a). A high reversibility of electron accumulation in the mesoporous semiconductor film gives rise to a symmetrical shape of the CV as the values of cathodic and anodic currents are comparable at each electrode potential (Bertoluzzi et al., 2013). While the CVs of films annealed at *T* ≥ 100°C virtually overlap at *E*_Ag/AgCl_ ≤ −0.3 V and feature a symmetrical shape, significantly lower current densities as well as a distorted shape are observed in the CV of a pristine film.

**FIGURE 2:**
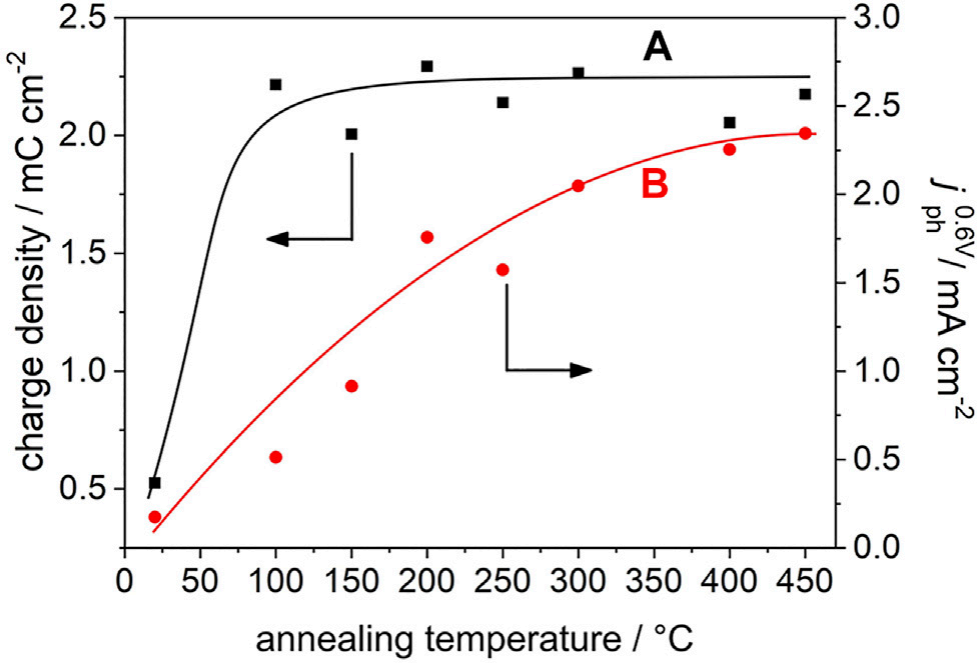
**(A)** Charge accumulated during the cathodic (i.e., negative-going) scan (in the absence of UV light) in the pristine and in the annealed electrodes (squares) as extracted from data in Figure 1A. **(B)** Saturated photocurrents recorded at *E*_Ag/AgCl_ = 0.6 V upon illumination with polychromatic UV/Vis light (circles) as extracted from data in Figure 1C. The lines are a guide to the eye.

All films are characterized by comparable specific surface areas and primary particle sizes [*d* = 14.5 nm, see Ref. (Rettenmaier et al., 2019)]. Importantly, CVs of all annealed films yield comparable current densities (Figure 1A). It has been shown that for mesoporous anatase TiO_2_ electrodes the capacitive current densities as well as the amount of accumulated charge (as extracted from the CVs) scales with the electrochemically active surface area (Berger et al., 2012a). Obviously, annealing at a temperature as low as 100°C imparts to the electrodes sufficient conductivity to electrochemically address—at least at sufficiently negative potentials—the whole nanoparticle network. Correspondingly, the accumulated charge (as determined from the cathodic scan) in TiO_2_ electrodes annealed at 100°C ≤ *T* ≤ 450°C is virtually constant (*Q* = 2.2 ± 0.1 Cg^−1^, Figure 2A).

In the pristine TiO_2_ electrode, significantly less charge is accumulated (*Q* = 0.5 Cg^−1^). This points (at comparable primary particle sizes) to a lower electrochemically active surface area and thus to limited interparticle charge transfer. Consequently, only a minor part of the films’ internal surface area contributes to the capacitive currents. Clearly, particle/particle contacts resulting from the sole drying of immobilized colloidal particles at room temperature do not impart sufficient conductivity to pristine TiO_2_ electrodes. The high resistance of the resulting pristine film gives rise 1) to the distorted shape of the CV and 2) to low current densities (Figure 1A). An annealing-induced modification of particle/particle contacts at temperatures as low as 100°C, however, imparts to the films sufficient conductivity to address the entire TiO_2_ film by electrochemical means (Rettenmaier et al., 2019).

Voltammetry is a powerful tool for studying the density of electrochemically active states in mesoporous semiconductor electrodes (Bisquert et al., 2008; Berger et al., 2012a). An in-depth analysis of the CVs of annealed electrodes allows to extract additional information on the energetic distribution of band gap states. The cathodic branches of the CVs feature in addition to an exponentially increasing current density at *E*_Ag/AgCl_ < −0.35 V (Figure 1A) a capacitive peak of low intensity at −0.35 V < *E*_Ag/AgCl_ < 0.1 V (Figure 1B). Whereas the exponential increase in current density has been assigned to the population of shallow traps located at the semiconductor/electrolyte interface (Bisquert et al., 2008), the capacitive peaks were attributed to the population of deep trap states located at grain boundaries (Lana-Villarreal et al., 2010; Jankulovska et al., 2012). Upon increasing the annealing temperature of the films, these peaks are shifted towards higher potentials (from *E*_Ag/AgCl_ = −0.20 V for a film annealed at 150°C to *E*_Ag/AgCl_ = 0.05 V for a film annealed at 450°C; Figure 1B). This shift can be associated with a modification of the energy of deep traps. Thermal annealing obviously induces changes in the atomic arrangement at grain boundaries and leads to an energetic stabilization of associated deep traps, which shift deeper into the band gap upon particle consolidation. From our experimental results we can not deduce conclusively, whether thermal annealing furthermore leads to a minor decrease of the intensity of capacitive peaks associated with deep traps. Due to the overlap of different capacitive contributions (resulting from traps at the solid/electrolyte interface and at solid/solid interfaces, respectively) we were not able to resolve any significant changes.

### Charge Separation Efficiency in Nanoparticle Electrodes

Photocurrents can be detected for semiconductor nanoparticle electrodes when the applied potential (Fermi level, *E*_F_, of the conducting substrate) is more positive than the potential corresponding to the conduction band edge (*E*_CB_) of the semiconductor (Berger et al., 2012a). For anatase TiO_2_ nanoparticle electrodes in 0.1 M HClO_4_, the position of the conduction band edge was estimated from photocurrent onset measurements in the absence of surface recombination and trapping at *E*_CB_ = −0.7 V vs. Ag/AgCl (3 M KCl) RE (Berger et al., 2012b). However, the photocurrent onset may significantly shift to more positive potentials due to electron-hole recombination, trapping of holes at surface defects and/or poor hole transfer to acceptors in solution (Bai et al., 2015). The detected photocurrent is thus a direct measure of the charge carrier separation efficiency in semiconductor electrodes.

The pristine and annealed anatase TiO_2_ nanoparticle electrodes were used as photoanodes. After each annealing step, CVs were recorded upon exposure of the film to UV light (Figure 1C). Methanol, a frequenly employed hole scavenger in photoelectrochemical studies (Jiang et al., 2001; Monllor-Satoca et al., 2011; Mesa et al., 2017), was employed to minimize surface recombination. At negative potentials (*E*_Ag/AgCl_ < −0.5 V), the current response of the electrodes resembles the one measured in the absence of UV light and results from capacitive dark currents (compare with Figure 1A) and the absence of significant photocurrents (Figure 1C). When scanning the electrode potential from the photocurrent onset potential towards more positive values, a linear increase of the photocurrent is observed for all annealing temperatures. At sufficiently positive potentials, a saturated photocurrent is reached (Figure 1C). Importantly, the saturated photocurrent (determined at *E*_Ag/AgCl_ = 0.6 V) increases almost linearly with annealing temperature in the range 100°C ≤ *T* ≤ 300°C and levels off at *T* = 400 and 450°C (Figures 1C, 2B).

Annealing temperature affects—in addition to the saturated photocurrent—also the onset of photocurrent saturation, which is observed in the potential range −0.25 V ≤ *E*_Ag/AgCl_ ≤ 0.10 V (Figure 1C). This potential shifts towards more positive values upon an increase of the annealing temperature from 100 to 450°C. The maximum photocurrent is attained at *E*_Ag/AgCl_ ≥ −0.25 V, *E*_Ag/AgCl_ ≥ 0.06 V and *E*_Ag/AgCl_ ≥ 0.10 V for a film annealed at 100, 300, or 450°C, respectively. This shift resembles the shift of the energy of deep trap states located at the grain boundaries (Figure 1B).

**SCHEME 1:**
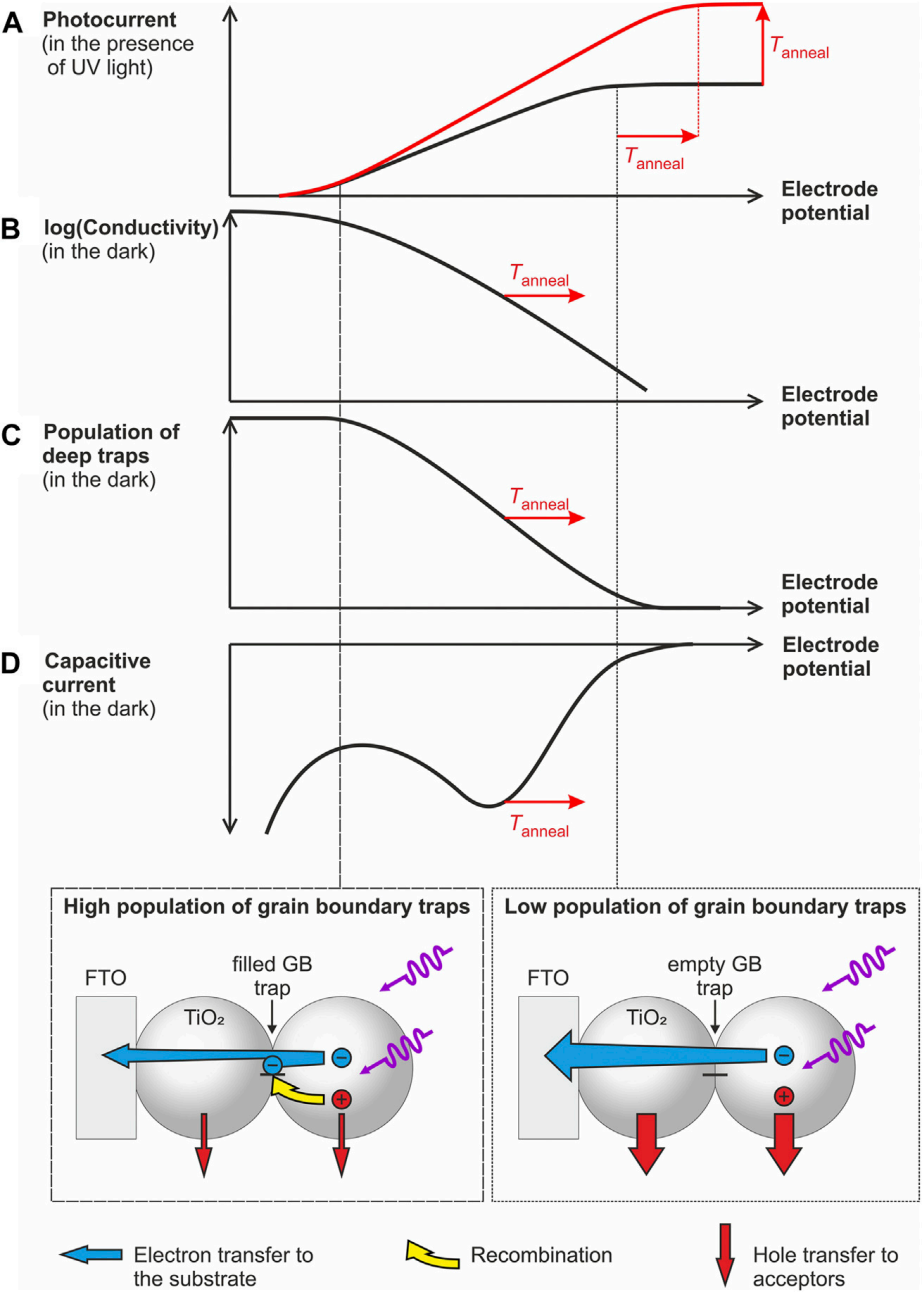
**(A–D)** Scheme highlighting the impact of the population of deep traps (as deduced from capacitive peaks in the dark voltammogram) on the electronic conductivity [as determined in Ref. (Rettenmaier et al., 2019)] and on the saturated photocurrent (i.e., the charge separation efficiency of photogenerated electrons and holes). Vertical lines indicate electrode potentials, where grain boundary traps will be highly populated (dashed line and scheme at the bottom, left) or (partially) depopulated (dotted line and scheme at the bottom, right).

The separation efficiency of photogenerated charge carriers (and thus the measured photocurrent) is the result of the kinetic competition between different processes including transport, transfer and recombination of charge carriers, which depend on electrode potential and thus on trap population. To rationalize the impact of deep traps and their population state on charge separation efficiency, we summarized the experimental findings from this and from our previous study (Rettenmaier et al., 2019) in Scheme 1.

For all electrodes investigated, we observe that the maximum obtainable photocurrent (Scheme 1A) is reached once deep trap states are mostly depopulated (Scheme 1C) as inferred from voltammetric measurements in the dark (Scheme 1D). This points to the strong impact of the population state of deep traps (Scheme 1C) on charge carrier separation. A possible explanation for this dependency is the involvement of deep traps in charge carrier recombination. Apparently, populated deep traps act as more efficient recombination centers than empty ones (Scheme 1, bottom). The increased recombination rate possibly results from the action of local electrostatic fields resulting from an uncompensated negative charge thus attracting photogenerated holes (Scheme 1, bottom). Indeed one may envisage—especially at high trap population—an incomplete charge compensation by counter ions from solution (such as e.g., H^+^) (Jiménez et al., 2016) at the buried interface of the grain boundaries. Consequently, photocurrents saturate at potentials, at which deep traps are depopulated. In such a case, the branching ratio between hole transfer to water or methanol and electron-hole recombination at populated grain boundary sites is increased and the photocurrent reaches its maximum value. Due to the energetic stabilization of deep traps upon thermal processing, this situation is reached at more positive potentials for annealed electrodes (Scheme 1A).

In addition to recombination, changes in the population state of deep traps are also associated with differences in conductivity (Scheme 1B) (Rettenmaier et al., 2019; Zhang et al., 2014). The electronic conductivity of the films increases exponentially in a potential range where deep traps get electrochemically populated and reaches an annealing temperature-independent value only at very negative potentials i.e., at potentials at which all deep traps are filled (Schemes 1B–D) (Rettenmaier et al., 2019). Such negative potentials corresponding to very high levels of electron accumulation are, however, not relevant for photoelectrodes assuring an efficient extraction of photogenerated electrons and generating thus measurable photocurrents (Schemes 1A,C).

The onset potential of the conductivity increase shifts positive upon increasing annealing temperature (Scheme 1B) in line with the displacement of the pair of capacitive peaks associated with deep trap states (Scheme 1D). This results—at a given electrode potential and under conditions, where deep traps are only partially populated—in a higher electronic conductivity for annealed electrodes. Consequently, photogenerated electrons are transported faster to the conductive substrate, which may explain the increase of the saturated photocurrent upon particle consolidation (Scheme 1A).

**SCHEME 2:**
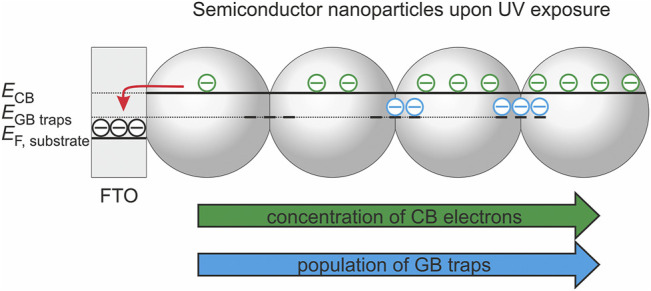
Population of conduction band (CB) states and of grain boundary (GB) traps under photostationary conditions.

For mesoporous nanoparticle electrodes there exists typically a wide potential range where the conductivity of the semiconductor film is high enough to allow for a homogeneous charging of the electrode and thus for a homogeneous displacement of the semiconductor Fermi level with respect to the conduction band as long as the band edges are pinned (Bisquert et al., 2008; Berger et al., 2012a). Consequently, a homogeneous equilibrium occupancy of band-gap states corresponding to the externally controlled substrate potential can be established. However, upon UV exposure and at sufficiently positive potentials (i.e., potentials more positive than the onset potential for charge accumulation in the dark), a gradient of the carrier concentration (and thus the Fermi level, *E*_F_) is established within the semiconductor film (Scheme 2) (Berger et al., 2012a). In direct vicinity of the conductive substrate (FTO), the Fermi level of the TiO_2_ nanoparticles possibly equilibrates with the potential applied to the substrate. However, the Fermi level of the TiO_2_ nanoparticles, which are exposed to UV light, is shifted towards more negative potentials as the photogenerated holes are at least partly transferred to solution species whereas the electrons accumulate in the semiconductor. Consequently, a gradient of electron concentration arises along the TiO_2_ film thickness. At electrode potentials, where electrochemically addressable deep traps are virtually depopulated in the dark, they may be at least partially populated upon UV excitation (Scheme 2) (Zhang et al., 2014). The gradient in the occupancy of deep traps—which will vary according to the annealing temperature-dependent trap energy—will give rise to local variations of the electron conductivity and of the recombination rate. The overall charge separation efficiency (as tracked by the photocurrent) represents, therefore, an integral electrode property resulting from the kinetic competition of generation, transport, recombination, and interfacial transfer of charge carriers.

### Charge Separation Efficiency in Nanoparticle Films

**FIGURE 3:**
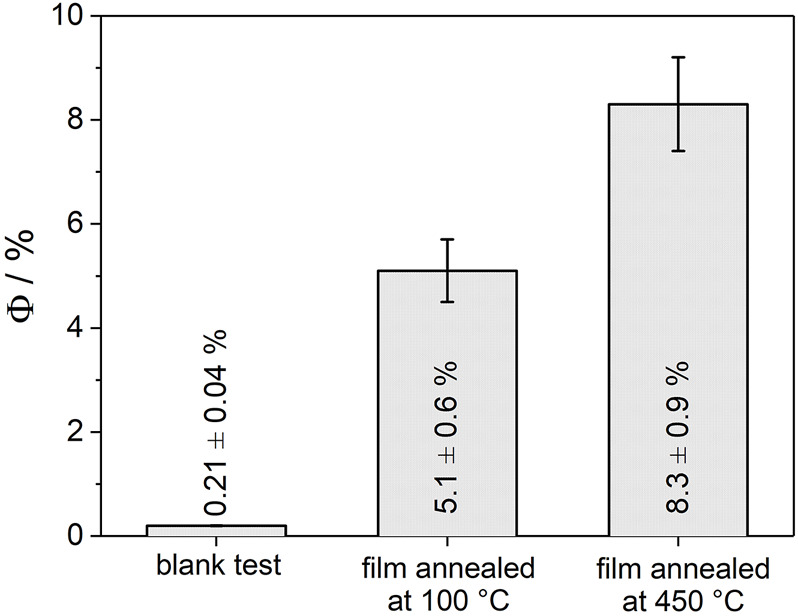
Apparent quantum yield (*Φ*) for H_2_O_2_ decomposition on immobilized anatase TiO_2_ nanoparticle layers (TiO_2_ mass: *m* = 1.5 mg, geometrical area of the film: 1.8 cm^2^, glass substrate) upon UV-exposure (*P* = 10 mW cm^−2^, *λ* = 360 nm). Films were annealed at 100 or 450°C, respectively. The reaction batch was cooled by an ice bath. Two independent experiments using freshly prepared films were performed for each annealing temperature. Blank tests using uncovered glass substrates (i.e., in the absence of the TiO_2_ layer) were conducted under otherwise identical conditions. Test reagent: 4.0 mM H_2_O_2_ aqueous solution purged with N_2_.

The beneficial effect of particle consolidation upon thermal annealing is also reflected in the photoactivity of anatase TiO_2_ nanoparticles immobilized on glass substrates. The apparent quantum yield for H_2_O_2_ decomposition (at *λ* = 360 nm) accounts for *Φ* = 5.1 ± 0.6% and *Φ* = 8.3 ± 0.9% for films annealed at 100 and 450°C, respectively (Figure 3). In the absence of the TiO_2_ layer, but under otherwise identical experimental conditions, the quantum yield accounts for 0.21 ± 0.04%. After 6 h of UV exposure a H_2_O_2_ conversion of 14 and 22% was observed for films annealed at 100 and 450°C, respectively. These results are fully in line with previous observations on the impact of aggregation and the associated formation of extended photocatalyst structures on charge separation (Wang et al., 2006; Lakshminarasimhan et al., 2008; Bian et al., 2012; Park et al., 2013; Wang et al., 2018). The promotion of charge carrier separation was attributed to the creation of additional pathways involving interparticle charge transfer and thus requiring sufficient conductivity. The beneficial effect of thermal annealing as observed in the present study can therefore be attributed to the increase of electronic conductivity upon particle consolidation and preservation of the active surface area (Rettenmaier et al., 2019). As highlighted for nanoparticle electrodes, the difference in film conductivity is especially pronounced under experimental conditions where deep traps at grain boundaries are not fully occupied (Rettenmaier et al., 2019). This will be the case in photocatalytic reactions under photostationary conditions, where an electron acceptor (such as H_2_O_2_) (Hirakawa and Nosaka, 2002; Friedmann et al., 2010) is typically added to the solution.

## General Discussion

The complementary investigation of immobilized semiconductor nanoparticle ensembles in the form of films and electrodes clearly highlights the strong impact of deep electron traps on charge separation in aggregated nanoparticle structures. By the careful choice of processing conditions (comprising the high temperature pretreatment of the nanoparticle powder at *T* = 600°C prior to slurry preparation, layer deposition and annealing at *T* ≤ 450°C), different degrees of particle consolidation can be obtained upon preservation of the structural properties of the nanoparticle films. Indeed, rather than inducing significant changes of the microstructure, such thermal processing is expected to lead to minor changes of the atomic arrangement at particle/particle contacts within the films, which we propose as the origin of the modified energetic distribution of deep trap states. Importantly, it has been shown for defect- and impurity-free TiO_2_ model grain boundaries that trap states at the solid/solid interface are associated with local perturbations of the electrostatic potential resulting from undercoordination of Ti sites, topological disruption and strain (Wallace and McKenna, 2014). It is feasible that the on-site electrostatic potentials of trap states at the particle/particle interface experience major modifications upon thermal energy input. We assume that particle/particle interface formation at low annealing temperatures leads to the build-up of local strain, which cannot be released at *T* ≤ 450°C.

The strong impact of the population state of deep traps on the dynamic response of photoelectrodes has been elucidated previously by employing periodic light perturbation experiments (Zhang et al., 2014). The dynamic photocurrent response of TiO_2_ nanotube arrays, where each nanotube consisted of a mosaic of nanoscopic anatase crystallites, was rationalized in terms of electron diffusion to the conducting substrate and the kinetics of trapping/ detrapping from deep trap states. Importantly, an increase of the characteristic rise time of the photocurrent, which was measured as a function of electrode potential, was observed upon the depopulation of deep trap states.

Considering the application of nanoparticle-based functional materials, our results evidence that particle consolidation constitutes a means of manipulating the energy of deep traps and thus the (potential-dependent) probabilities for interparticle charge transfer and recombination at particle/particle contacts. From an analytical point of view, related studies highlight the suitability of electrochemical methods to characterize photocatalyst materials *in situ* and to contribute to a microscopic understanding of macroscopic functional properties of nanoparticle-based materials.

## Conclusion

In conclusion, our results highlight the strong impact of energy and population of deep traps on charge separation efficiency and thus photoactivity of anatase TiO_2_ nanoparticle films. Nanoparticle consolidation upon thermal annealing leads to an energetic stabilization of deep traps. The partial population of these traps during electrode / film operation influences the branching ratio between charge separation and electron/hole recombination due to (at least) two different processes: 1) the increase of the electronic conductivity upon trap population and 2) the presence of local electrostatic fields at grain boundaries attracting photogenerated holes. Thermal annealing of nanoparticle films at temperatures as low as 100°C (and up to 450°C) favors charge separation and leads under the investigated experimental conditions to higher photocurrents and apparent quantum yields.

## Data Availability

The original contributions presented in the study are included in the article/supplementary material, further inquiries can be directed to the corresponding author.
